# Correlatıon between PET-CT uptake values and pathologıcaly features ın head and neck cancer

**DOI:** 10.1007/s00405-025-09228-9

**Published:** 2025-03-13

**Authors:** Atakan Sarıgül, Vahit Mutlu

**Affiliations:** Otolaryngology Department, Atatürk University Research Hospital, Yakutiye, 25240 Erzurum, Turkey

**Keywords:** HNSCC, SUV-Max, PET-CT, Perineural invasion, Lymphovascular invasion, Metastasis, Prognosis

## Abstract

**Objectives:**

To evaluate the correlation between SUV-Max values and pathological outcomes in head and neck squamous cell carcinoma (HNSCC) and determine the predictive power of SUV-Max for disease prognosis.

**Data sources:**

Retrospective analysis of medical records and PET-CT imaging results from patients diagnosed with HNSCC at our institution between 2014 and 2023.

**Review methods:**

Examination of SUV-Max values from F18-FDG PET-CT scans and their association with pathological findings such as perineural invasion, lymphovascular invasion, and neck lymph node metastasis. Statistical analysis was conducted to establish cutoff values and assess the significance of correlations.

**Results:**

Our study identified significant cutoff values for PET-CT SUV-Max that correlate with the pathological features of head and neck cancer. For primary tumors, a SUV-Max cutoff of 14.71 predicted neck metastasis with a sensitivity of 67.6% and specificity of 64.2%, demonstrating moderate diagnostic accuracy with an AUC of 0.648. Perineural invasion was optimally predicted at a cutoff of 13.28, with a sensitivity of 74%, specificity of 67.3%, and an AUC of 0.728. Similarly, a cutoff of 13.28 for lymphovascular invasion yielded a sensitivity and specificity of 63%, with an AUC of 0.628. Additionally, neck lymph node metastasis was effectively assessed with a SUV-Max cutoff of 2.74, achieving a sensitivity of 62.2%, specificity of 67%, and an AUC of 0.694. These cutoff values highlight the potential of SUV-Max in enhancing diagnostic precision for both primary tumors and lymph node assessments in head and neck oncology.

**Conclusion:**

SUV-Max values from PET-CT scans are significant predictors of pathological outcomes in HNSCC, aiding in the stratification of patient prognosis and guiding clinical decision-making.

## Introduction and aim

Head and neck cancers are the sixth most common type of cancer. If we consider head and neck cancers individually, they consist of 91% squamous cell, 7% adenocarcinoma, melanoma, and poorl defined tumors and 2% sarcomas [1]. The components of the head and neck squamous cell carcinoma (HNSCC) class include squamous cell carcinomas of the lip, oral cavity, oropharynx, and larynx. The incidence in the male population is four times higher than in females. Alcohol consumption, frequency, duration and amount of tobacco use are the main factors affecting the development of HNSCC [2]. Additionally, immunocompromised conditions, genetic predisposition, exposure to radiation and factors such as the Human Immunodeficiency Virus (HIV), Epstein-Barr Virus (EBV), and Human Papillomavirus (HPV) also negatively influence development [3]. Positron Emission Tomography (PET) scanning is one of the newly developed imaging tests used for evaluating tumor metastasis and primary involvement, preoperative assessment, recurrence monitoring. Studies have shown that it has superior sensitivity and specificity compared to computed tomography (CT) and magnetic resonance (MR) imaging in the evaluation of metastatic lymph nodes. Standard Maximum Uptake Volume (SUV-Max) is a quantitative parameter that assesses the activity of the mass in question by standardizing its glucose usage. There are numerous studies indicating that the SUV-Max value is related to the type of carcinoma, degree of differentiation, level of tumor proliferation and even survival [4–7].

In this study, the relationship between the preoperative SUV-Max value obtained from the F18-FDG PET scan and postoperative pathological outcomes such as neck metastasis, perineural invasion, lymphovascular invasion, metastasis in two or more lymph nodes, lymph node capsule invasion, and bilateral neck metastasis was investigated in patients who underwent surgery for the diagnosis of head and neck squamous carcinoma. The SUV-Max value of both the primary tumor and the lymph node in the neck was evaluated. A statistically significant cutoff value was determined for the factors with a significant relationship, which could assist the clinician.

## Methods

This is a retrospective study conducted on 160 patients diagnosed with squamous cell carcinoma of the lip, tongue and larynx, who underwent surgical treatment at our clinic and had an F18-FDG PET scan within the four months prior to surgery, meeting our inclusion criteria and not having the exclusion criteria. Inclusion criteria are being diagnosed with SCC between 2014 and 2023 having undergone a PET scan within four months prior to surgery having all preoperative and postoperative follow-ups conducted at our clinic. Exclusion criteria are diagnosis of additional malignancies previous history of chemotherapy or radiotherapy due to the current diagnosis, uncontrolled diabetes mellitus, active infection process, use of steroid treatment.

The radiological and pathological reports of the 160 patients who met the criteria were obtained from our hospital's automation system. The reports were deidentified and transferred to the Statistical Package for Social Science (SPSS) program for analysis.

## PET-CT protocol

Siemens Biograph 16 LSO HI-REZ integrated PET-CT scanner. After completing a minimum fasting period of 6 h, basal blood glucose levels were measured. Patients with Diabetes Mellitus (DM) who had blood glucose levels below 200 mg/dl were included in the scan. Those with higher levels had their values medically reduced to below 200 mg/dl. The patients’ weights were measured, and 1 mCi of 18 FDG per kilogram (ranging from 0.5 to 2 mCi) was administered intravenously one hour before the procedure. The scans were performed with the patients in a supine position, covering the area from the vertex to the thigh with a slice interval of 0.5 cm. The standard uptake volume and the maximum value (SUV-Max) of the areas with uptake were also evaluated to obtain data.

## Pathologic protocol

In patients diagnosed with SCC, neck dissection was performed on those suspected of having neck involvement or deemed in need of prophylactic cervical lymph node clearance. During surgery, the excised primary tumor and neck dissection material were separated as pathological specimens and delivered to the pathology clinic. In the pathological evaluation of the primary tumor, perineural invasion was reported in patients with SCC cells observed in the neural structures of the specimen, and lymphovascular invasion was reported in patients with SCC observed in lymphatic and vascular tissues. The malignancy level in the tumor was reported as well, moderately or poorly, based on the differentiation level of the malignant tissue. In patients who underwent neck dissection, lymph nodes with SCC in the cervical lymph nodes were reported as metastatic. The size, number and involved surgical neck areas of the metastatic lymph nodes were also reported. If SCC was observed in the capsule of the metastatic lymph node in the neck, it was reported as capsular invasion.

## SPSS protocol

The reports obtained were grouped in the SPSS program according to patient age, differentiation level, perineural invasion, lymphovascular invasion, neck metastasis, and lymph node capsule invasion status. Average SUV-Max values, minimum and maximum levels and standard deviation levels of these pathological data were obtained as descriptive statistical data. Additionally, the significance level of the correlation between these data and SUV-Max values was determined using the independent t-test. Statistically significant data (p < 0.05) were subjected to ROC analysis. Sensitivity and specificity values corresponding to each SUV-Max value were obtained and cut-off SUV-Max values were determined using the Youden index.

## Results

The study included 160 patients. All patients underwent primary tumor excision surgery, and 146 of them (91%) underwent neck dissection surgery (modified radical, selective, and superselective neck dissection). Of the patients, 142 (88%) were male and 18 (12%) were female. The average age was 62 (min: 28, max: 92). The distribution of the primary tumor SUV-Max value ranged from 2.92 to 45 (average: 11.9, Standard Deviation: 8.7). The neck lymph node SUV-Max distribution ranged from 1.28 to 18.58 (average: 3.4, Standard Deviation: 4). Primary tumor sizes ranged from 0.8 to 8 cm. Of the patients, 53 (33%) had lip SCC, 32 (20%) had tongue SCC, and 75 (47%) had laryngeal SCC. The primary tumor showed well differentiation in 52 patients (32%), moderate in 104 patients (65%), and poor in 4 patients (2.5%). Perineural invasion was found in 50 of the included patients (31%), and lymphovascular invasion was found in 54 patients (33%). According to the AJCC (American Joint of Cancer Committee) 2018 staging guidelines, 40 of the primary tumors were stage T1 (25%), 52 were T2 (32%), 32 were T3 (20%), and 36 were T4 (22.5%). Neck lymph node staging resulted in 109 patients at N0 (68%), 16 at N1 (10%), and 21 at N2 (22%). Of the 37 patients with neck lymph node metastasis, 13 (35%) had lymph node capsule invasion. In the PET-CT examination, neck metastasis was reported in 86 patients (53%). In 9 of the 74 patients (12%) who were reported to have no neck metastasis (47%), occult lymph node metastasis in the neck was observed. The sensitivity of the PET-CT examination for neck metastasis is 76%, and its specificity is 52% (Table 1).

**Table 1:** The breakdown and staging of the pathological data obtained in the study

		N	%
Location	Lip	53	33,125
	Tongue	32	20
	Larynx	75	46,875
	Supraglottic	25	15,625
	Glottic	50	31,25
Differentiation	Well	52	32,5
	Moderately	104	65
	Poor	4	2,5
Perineural Invasion	Yes	50	31,25
	No	110	68,75
Lymphovasculary Invasion	Yes	54	33,75
	No	106	66,25
T Stage (AJCC, TNM)	1	40	25
	2	52	32,5
	3	32	20
	4	36	22,5
N Stage (AJCC, TNM)	0	109	74,65
	1	16	10,95
	2	21	14,4
Neck Dissection	Yes	146	91,25
	No	14	8,75
LN Capsulary Invasion	Yes	13	35,13
	No	24	64,87
Bilaterality	Yes	13	35,13
	No	24	64,87
Neck Involvement	Yes	37	25,34
	No	109	74,66
PET/CT' Neck Involvement	Yes	86	53,75
	No	74	46,25

**Fig. 1:**
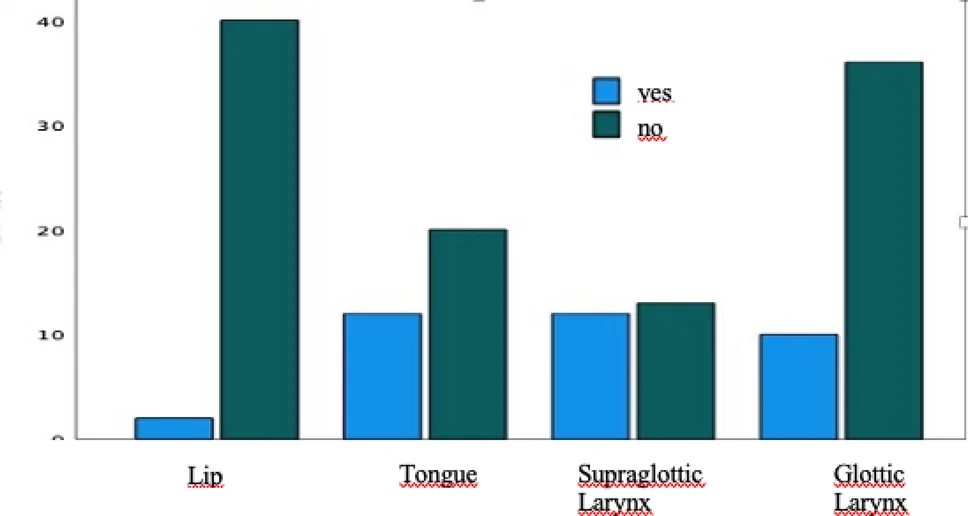
Relation between primary tumor location and neck metastasis status

In patients diagnosed with lip SCC, 2 (3.8%) had neck metastasis in their pathology reports, in tongue SCC patients, 12 (37.5%) had it, in supraglottic larynx SCC patients, 12 (48%) had it and in larynx SCC patients, 11 (22%) had it. Neck metastasis in patients with supraglottic larynx and tongue SCC is significantly higher compared to patients diagnosed with lip and glottic larynx SCC (p = 0.001) (Fig. 1).

**Fig. 2:**
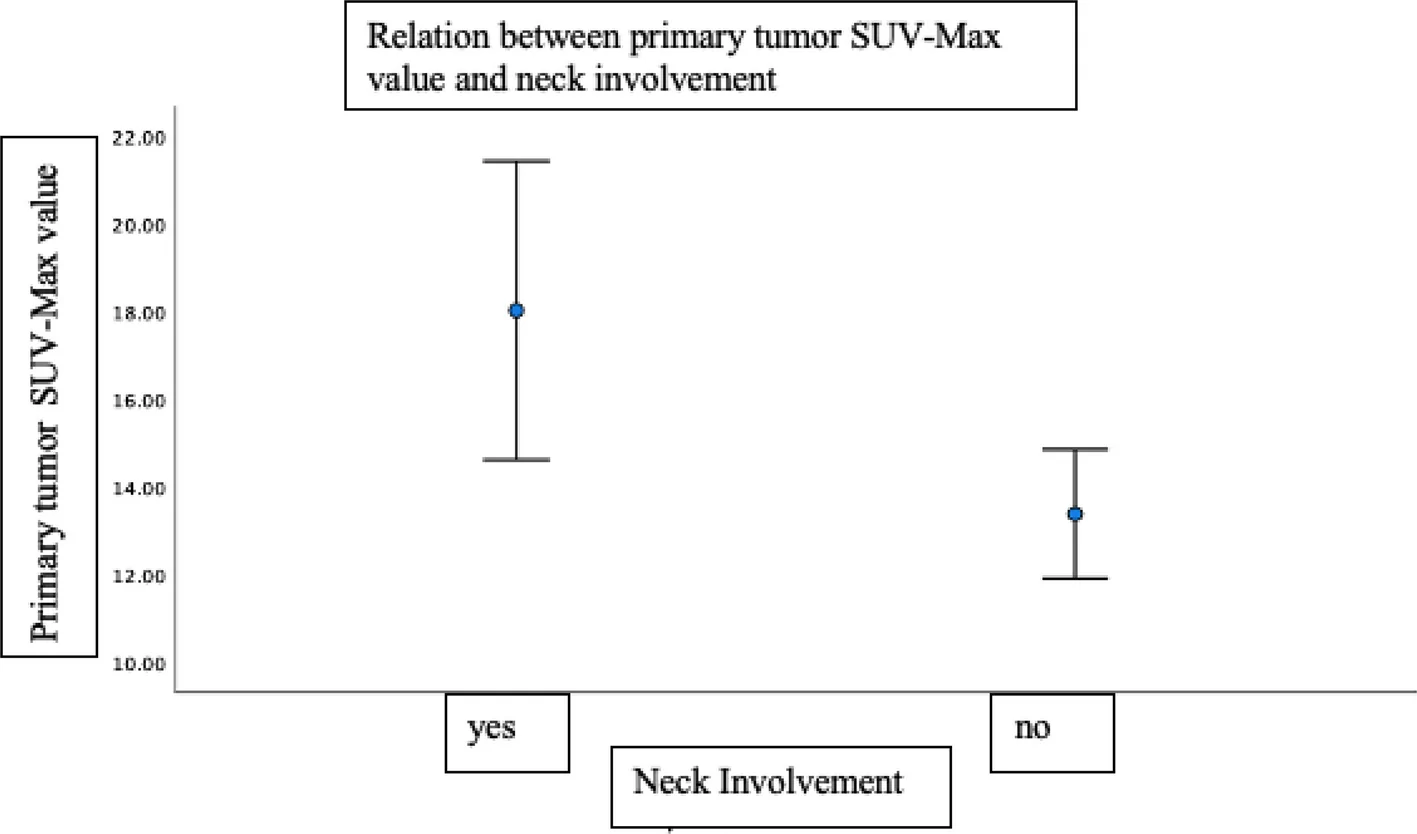
Relation between primary tumor SUV-Max value and neck metastasis

**Fig. 3:**
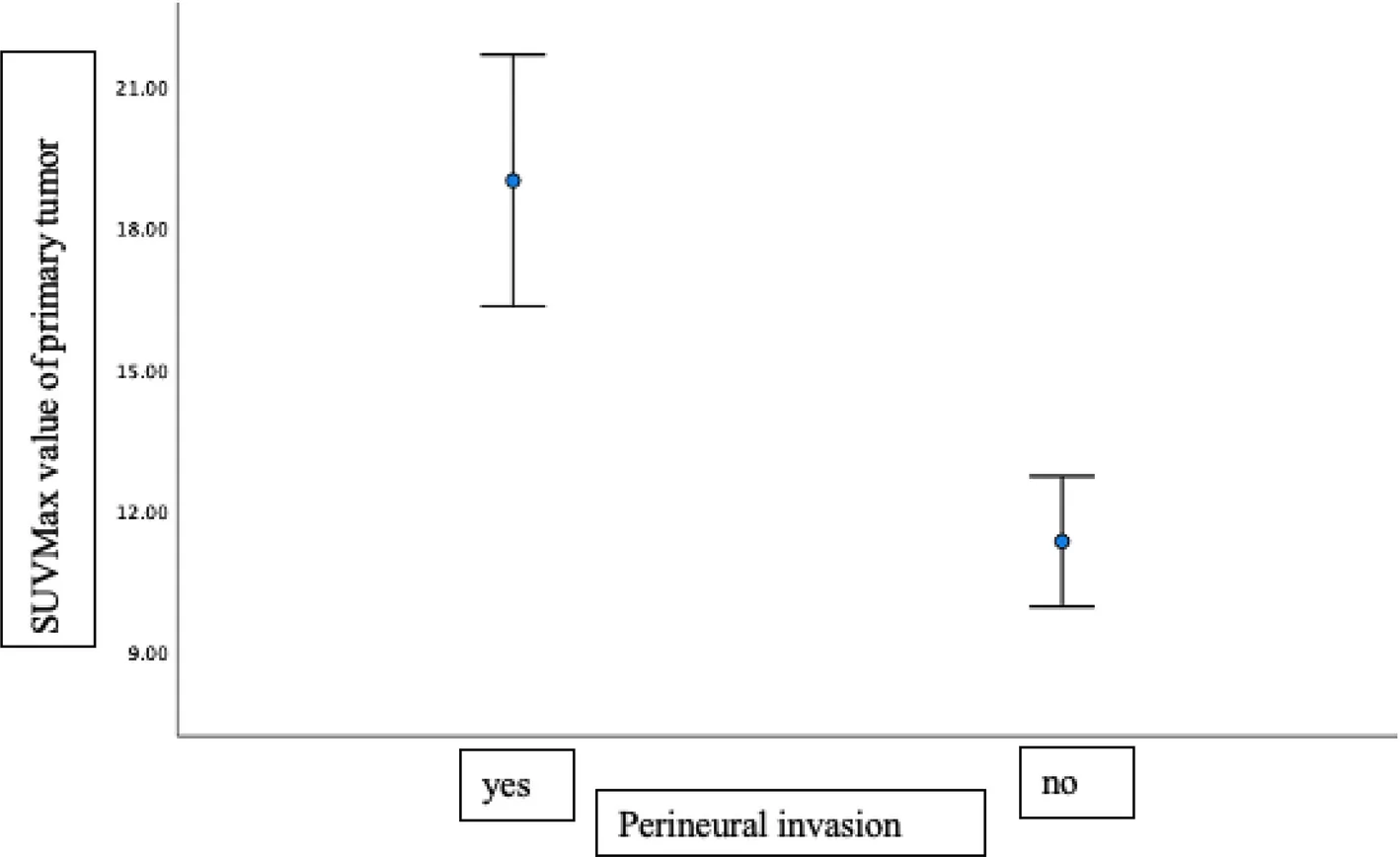
Relation between primary tumor SUV-Max value and perineural invasion

**Fig. 4:**
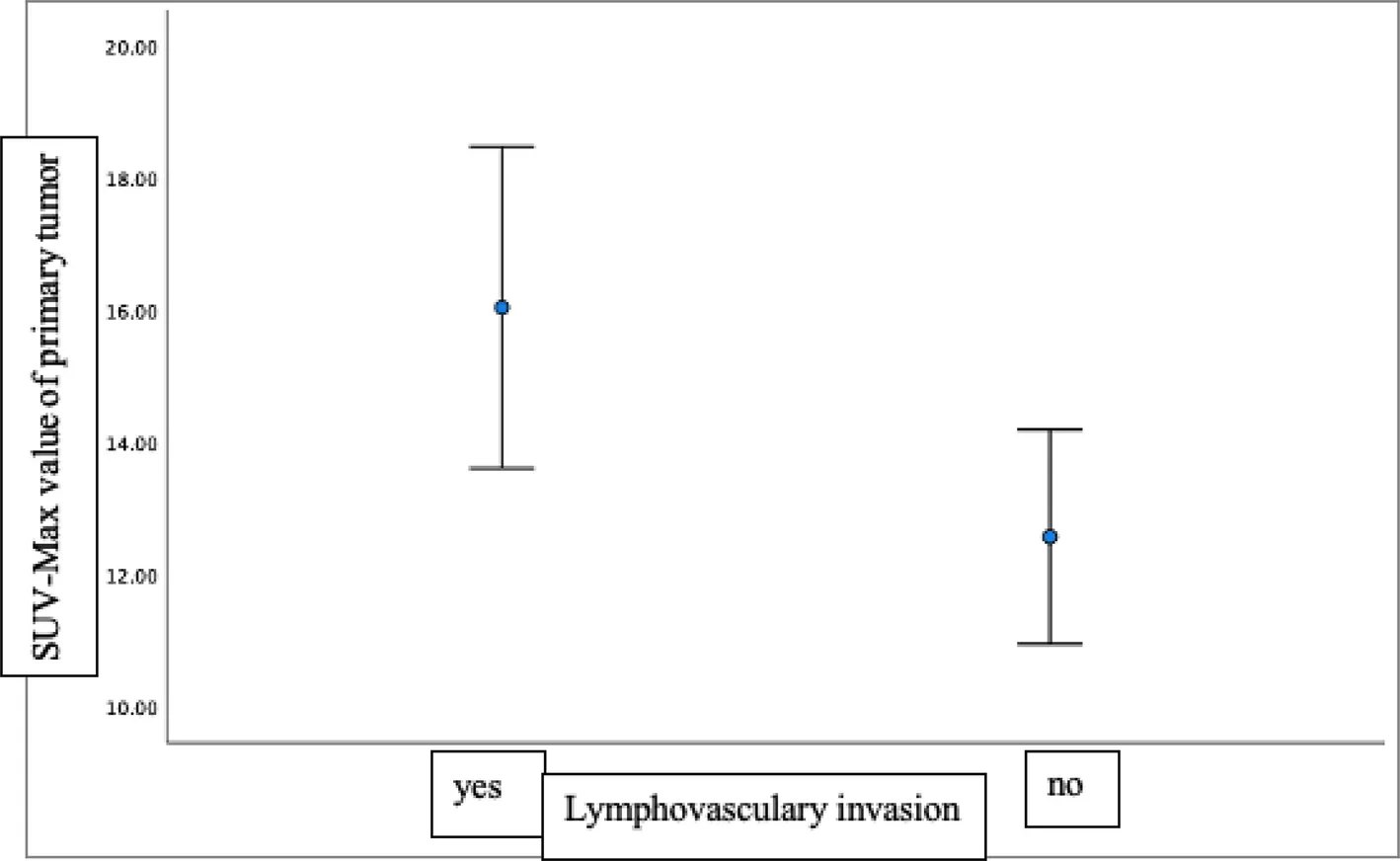
Relation between primary tumor SUV-Max value and lymphovasculary invasion

The average SUV-Max value of the primary tumor in patients with neck metastasis is 18.1, while in patients without neck metastasis, it is 13.39. The average SUV-Max value in patients with pathological neck lymph node metastasis is significantly higher compared to those without neck metastasis (p = 0.004). The average SUV-Max value of the primary tumor in 50 patients with perineural invasion is 18.99 (SD: 9.38), while it is 11.34 (SD: 7.3) in 110 patients without perineural invasion. The difference in SUV-Max values between these two groups is statistically significant (p < 0.01). The average SUV-Max value of the primary tumor in 54 patients with lymphovascular invasion is 16.04 (SD: 8.92), and in 106 patients without lymphovascular invasion, it is 12.56 (SD: 8.44). Similarly, the average primary SUV-Max value in patients with lymphovascular invasion is significantly higher than in those without it. Lastly, the average primary tumor SUV-Max value in 21 patients with invasion of two or more lymph nodes is 19.62 (SD: 8.95), compared to 13.79 (SD: 8.34) in 125 patients with no lymph node or only one lymph node metastasis. Thus, patients with metastasis in two or more lymph nodes have significantly higher primary tumor SUV-Max values compared to others (p = 0.005) (Figs. 2, 3, 4).

**Fig. 5:**
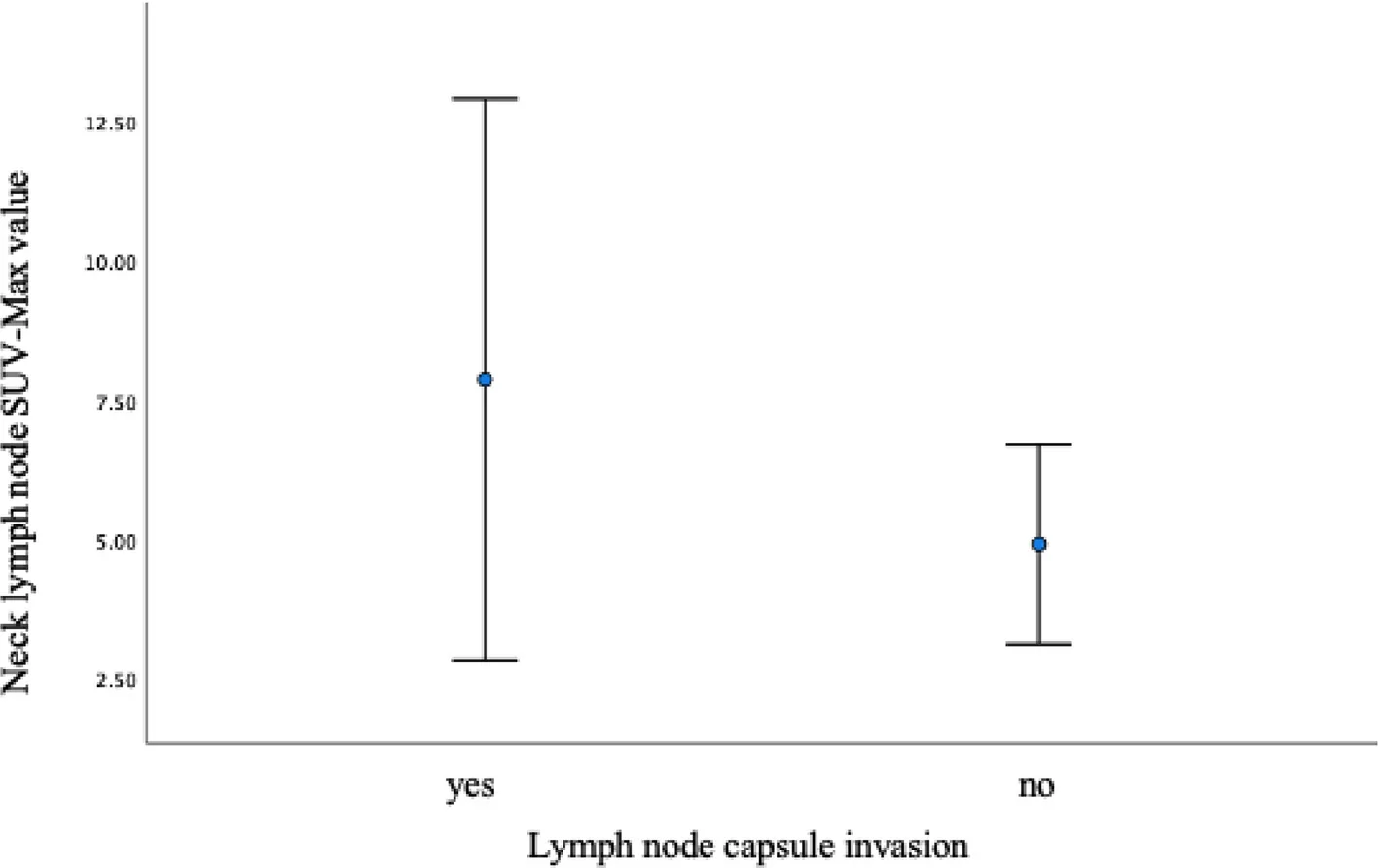
Relation between primary tumor SUV-Max value and lymph node capsule invasion

When comparing the PET-CT SUV-Max values of the neck lymph nodes with pathology data: the average SUV-Max value of the neck lymph node in 37 patients with neck metastasis is 5.64 (SD: 6), while in 110 patients without neck metastasis, it is reported as 1.9 (SD: 2.24). The difference in SUV-Max values between these two groups is statistically significant (p < 0.001). The average neck lymph node SUV-Max value in 21 patients with two or more lymph nodes is 7.52 (SD: 6.58), while it is 2.13 (SD: 2.66) in the other patients. The difference in the average SUV-Max value between these two groups is statistically significant (p < 0.001). Lastly, the average lymph node SUV-Max value in 14 patients with lymph node capsule invasion is 7.87 (SD: 2.31), compared to 4.91 (SD: 0.8) in 23 patients without capsule invasion. The average SUV-Max value of the neck lymph nodes in patients with lymph node capsule invasion is significantly higher than in those without it (p < 0.001) (Fig. 5).

When examining the relationship between bilateral involvement and primary tumor and neck lymph node SUV-Max values, the average primary tumor SUV-Max value in 13 patients with bilateral neck involvement is 21.62 (SD: 9.6), and the average neck lymph node SUV-Max value is 7.21 (SD: 11.26). In 18 patients without bilateral involvement, the average primary tumor SUV-Max value is 17.04 (SD: 11.26), and the average neck lymph node SUV-Max value is 5.94 (SD: 5.96). No significant difference was observed between the two groups in terms of either primary tumor or neck lymph node SUV-Max values (p > 0.05).

All conditions with statistically significant relationships were subjected to Receiver Operating Characteristic (ROC) analysis to test the correlation strength between the SUV-Max value and pathological data and a cutoff value was determined for each pathological datum. First, the relationship between the primary tumor SUV-Max value results and pathological outcomes will be described, followed by the relationship between neck lymph node SUV-Max value and pathological results.

**Fig. 6:**
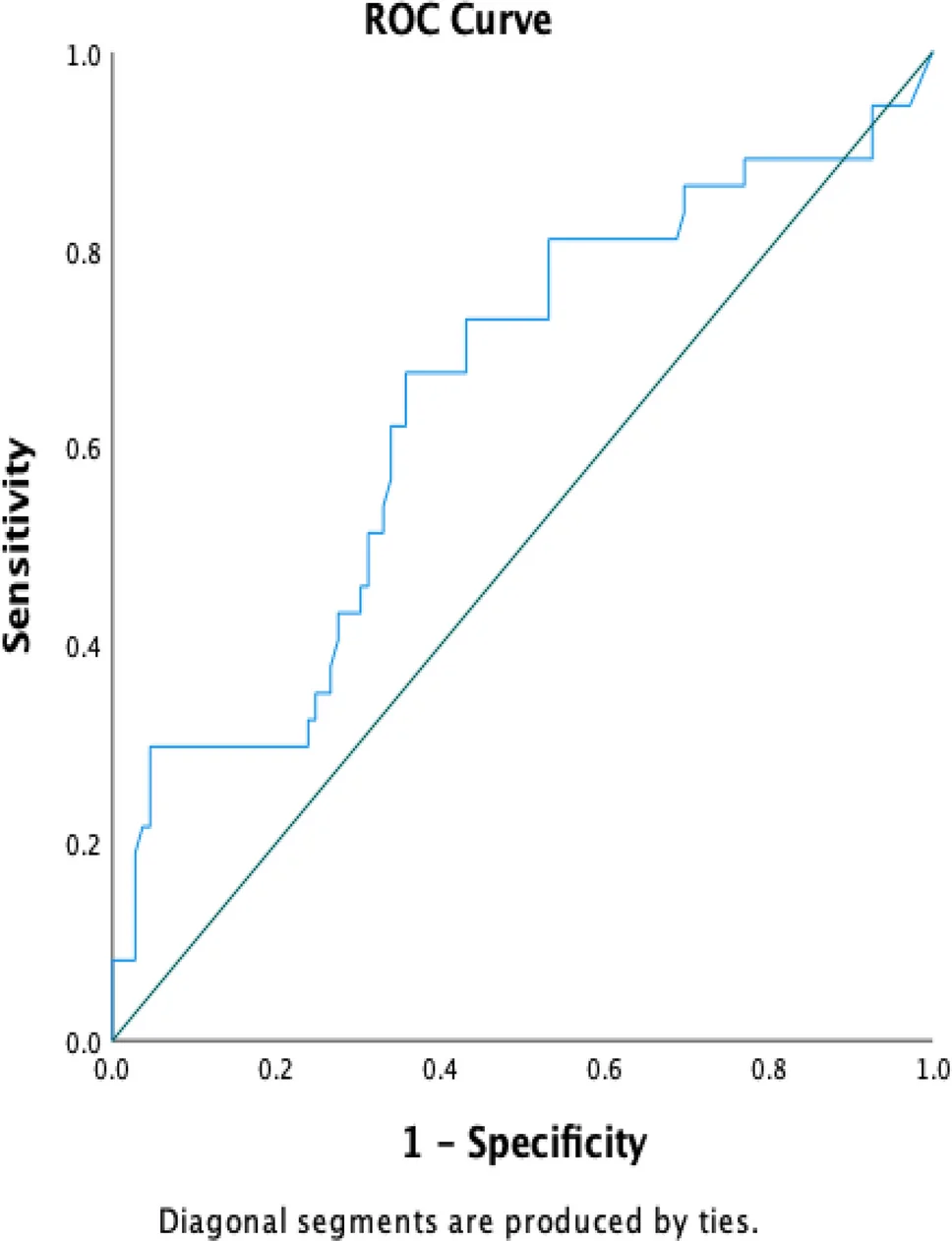
ROC curve of relation between primary tumor SUV-Max value and neck metastasis

Initially, the presence of metastatic lymph nodes in the neck was evaluated with the primary tumor SUV-Max value, resulting in an AUC (Area Under Curve) of 0.648 on the ROC curve. This AUC value indicates that the relationship between these two conditions is statistically significant at a moderate level. The cutoff value for neck metastasis, according to the Youden index table, has been determined as 14.71. For this value, the sensitivity is calculated as 67.6%, and the specificity as 64.2% (Table 2, Fig. 6).

**Table 2:** Relation between primary tumor SUV-Max value and neck involvement

	Cut-off	Sensitivity (%)	Specifity (%)	AUC
Primary tumor SUV-Max	14.71	67.6	64.2	0.648

**Fig. 7:**
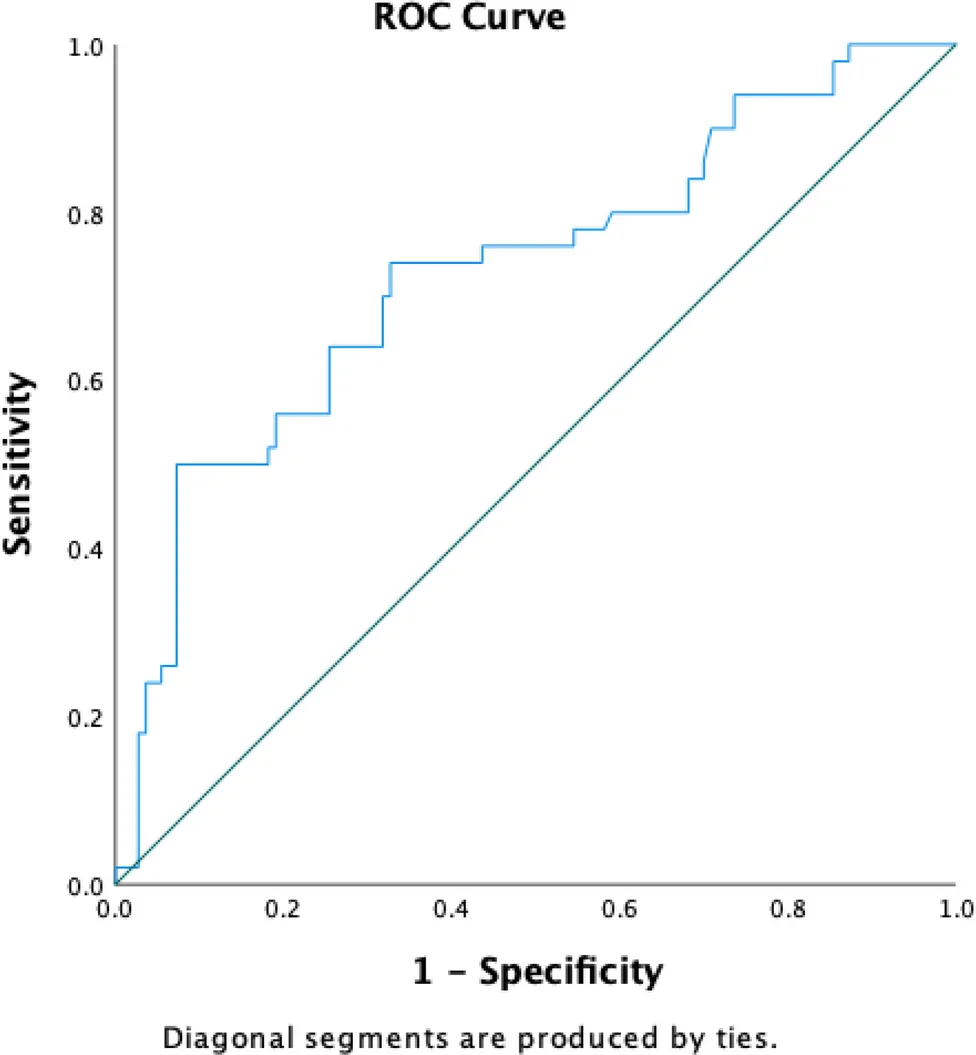
ROC curve of relation between primary tumor SUV-Max value and perineural invasion

Examining the relationship between the primary tumor SUV-Max value and the presence of perineural invasion resulted in an AUC (Area Under Curve) of 0.728 on the ROC curve. Consequently, we can say that the relationship between perineural invasion and the primary tumor SUV-Max value is statistically significant at a high level. The cutoff value has been determined to be 13.28. Based on this value, the sensitivity for the condition of perineural invasion is 74%, and the specificity is 67% (Table 3, Fig. 7).

**Table 3:** Relation between primary tumor SUV-Max value and perineural invasion

	Cut-off	Sensitivity (%)	Specifity (%)	AUC
PET Primary SUV-Max value	13.28	74	67.3	0.728

**Fig. 8:**
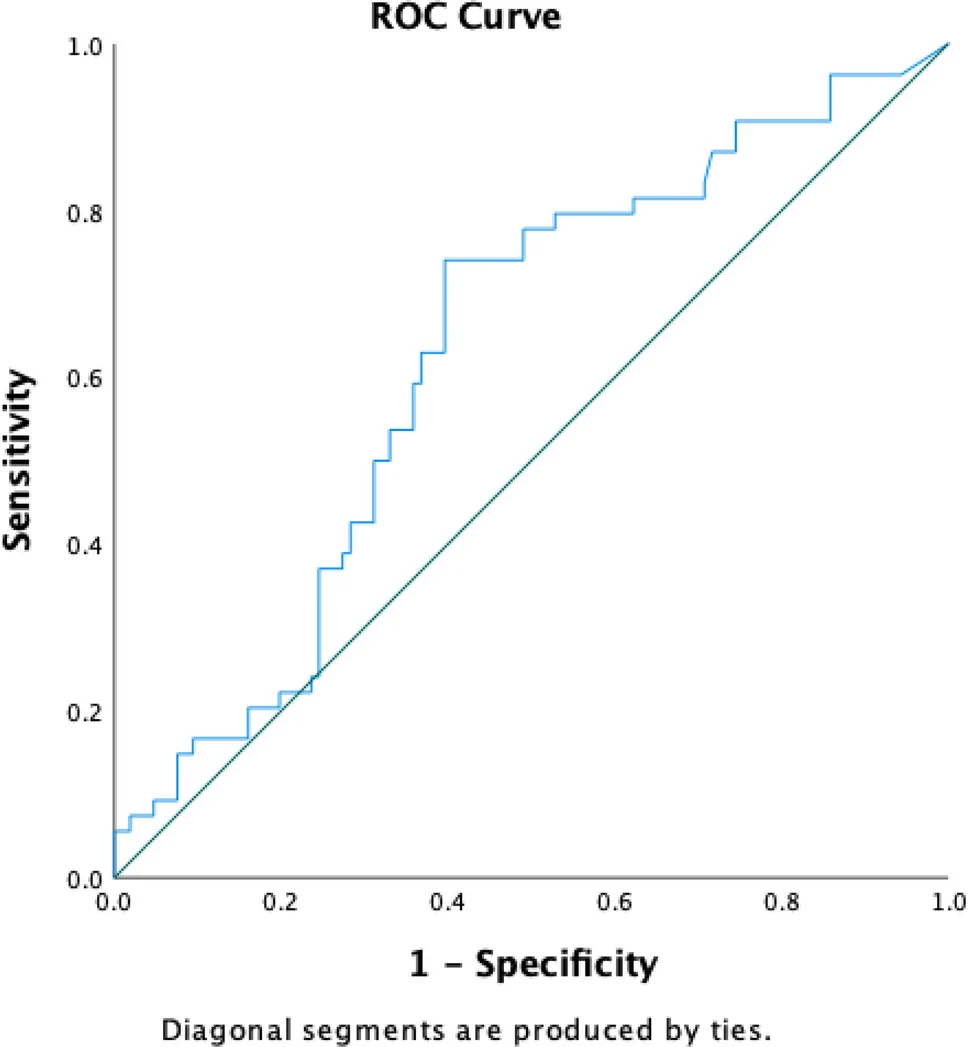
ROC curve of relation between primary tumor SUV-Max value and lymphovasculary invasion

The relationship between the primary tumor SUV-Max value and lymphovascular invasion was analyzed and resulted in an AUC (Area Under Curve) of 0.628 on the ROC curve. This indicates that the relationship between the SUV-Max value and lymphovascular invasion is statistically significant at a moderate level. The most useful cutoff value, like in the case of perineural invasion, has been determined to be 13.28. Accepting this value, the sensitivity is found to be 63% and the specificity 63% (Table 4, Fig. 8).

**Table 4:** Relation between primary tumor SUV-Max value and lymphovasculary invasion

	Cut-off	Sensitivity(%)	Specifity (%)	AUC
Primary tumor SUV-Max value	13.28	63	63	0.628

**Fig. 9:**
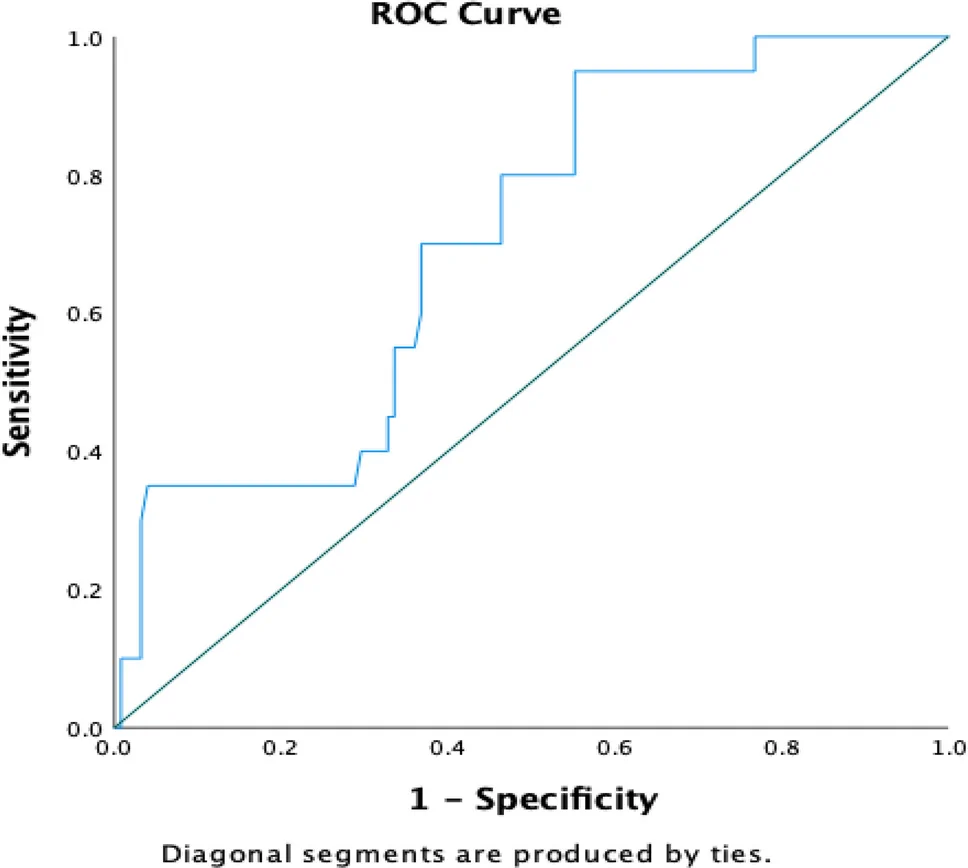
ROC curve of relation between primary tumor SUV-Max value and two or more neck lymph node metastasis

The last pathological datum tested for the primary tumor was the involvement of two or more lymph nodes. When evaluating the relationship with the primary tumor SUV-Max value, the resulting ROC curve had an AUC (Area Under Curve) of 0.704. This result indicates a strong relationship between the mentioned two variables. According to the Youden index, the best cutoff value was determined to be 14.99. In this case, the sensitivity is 70% and the specificity is 63.2% (Table 5, Fig. 9.

**Table 5:** Relation between primary tumor SUV-Max value and two or more neck lymph node metastasis

	Cut-off	Sensitivity (%)	Specifity (%)	AUC
SUV-Max	14.99	70	63.2	0.704

**Fig. 10:**
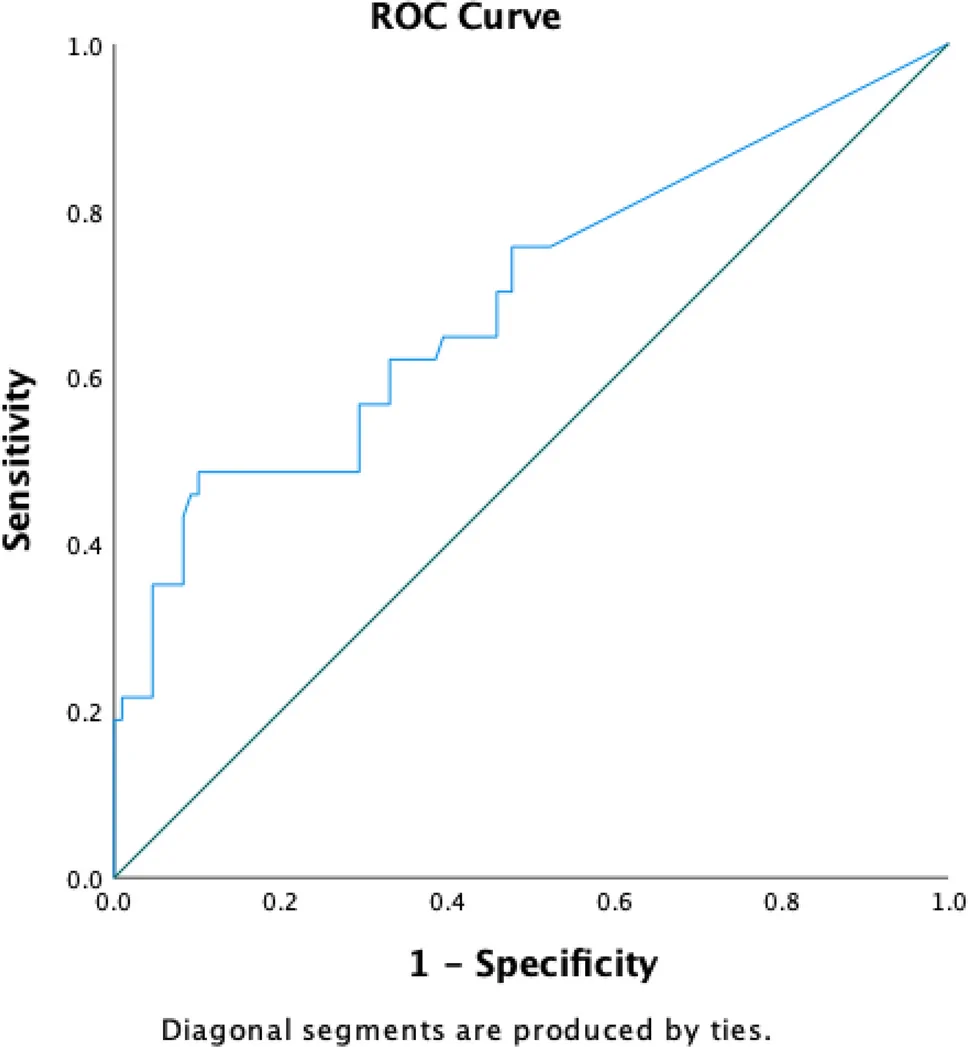
ROC curve of relation between neck lymph node SUV-Max value and neck metastasis

Following the primary tumor data, an assessment of the neck lymph nodes was also conducted. When examining the relationship between the neck lymph node SUV-Max value and neck involvement, the ROC curve resulted in an AUC (Area Under Curve) of 0.694. This indicates that the relationship between these two conditions is statistically significant at a moderate level, and the cutoff value for the neck lymph node has been determined to be 2.74, with a sensitivity of 62.2% and specificity of 67% (Table 6, Fig. 10).

**Table 6:** Relation between neck lymph node SUV-Max value and neck metastasis

	Cut-off	Sensitivity (%)	Specifity (%)	AUC
SUV-Max	2.74	62.2	67	0.694

**Fig. 11:**
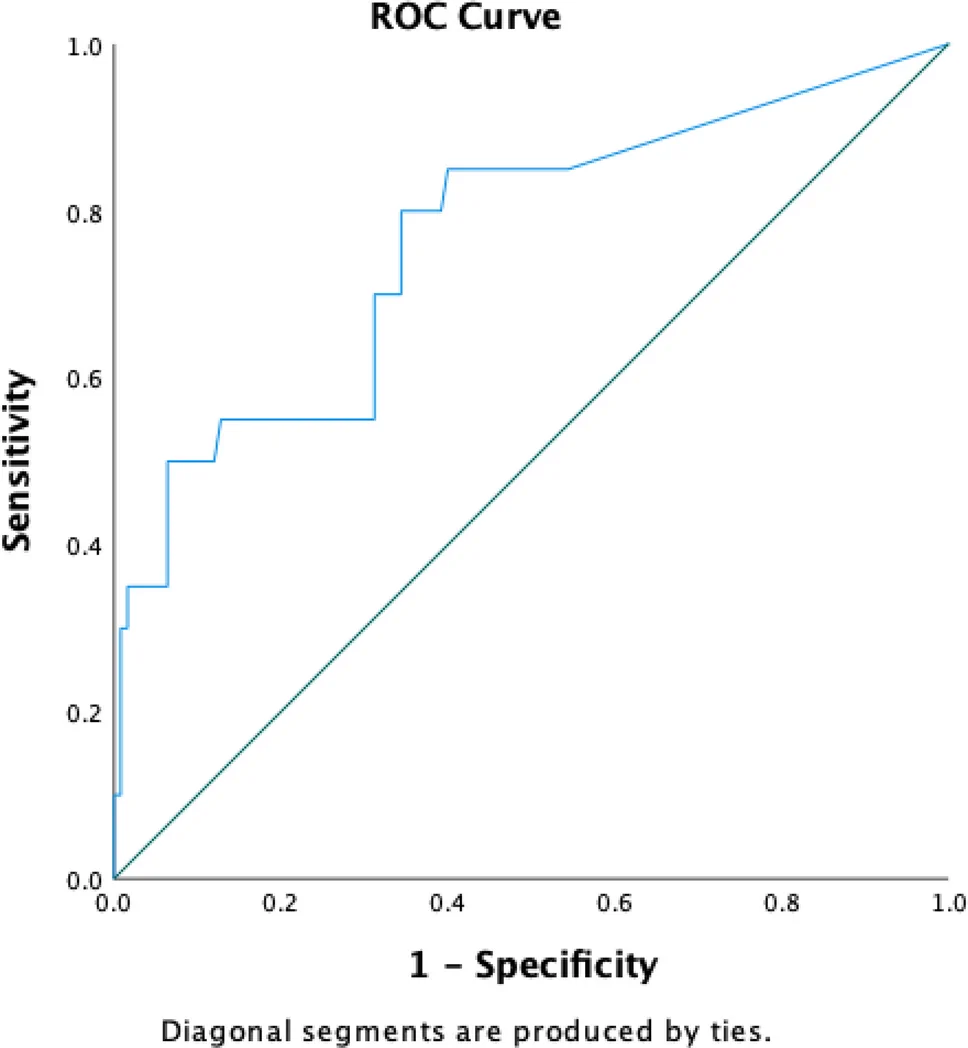
ROC curve of relation between neck lymph node SUV-Max value and two or more lymph node metastasis status

Subsequently, the relationship between the neck lymph node SUV-Max value and the involvement of two or more lymph nodes was analyzed. The ROC curve yielded an AUC (Area Under Curve) of 0.765. This result indicates a good level of relationship between the involvement of two or more lymph nodes and the neck lymph node SUV-Max value. The determined cutoff value is 3.22, with a sensitivity of 70% and a specificity of 68.8% (Table 7, Fig. 11).

**Table 7:** Relation between neck lymph node SUV-Max value and two or more lymph node metastasis status

	Cut-off	Sensitivity (%)	Specifity (%)	AUC
SUV-Max	3.22	70	68,8	0,765

**Fig. 12:**
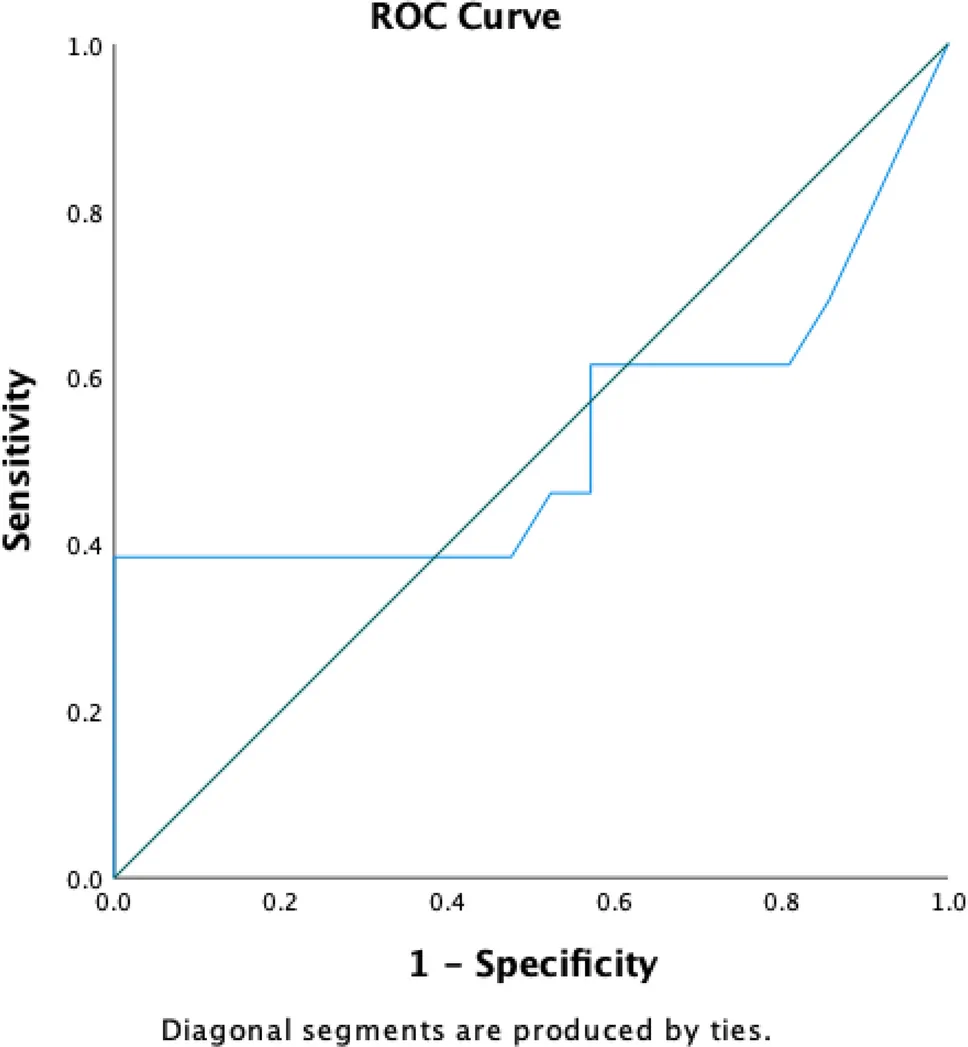
Relation between neck lymph node SUV-Max value and lymph node capsule invasion

The last pathological data tested was the relationship between lymph node capsule invasion and neck lymph node SUV-Max value. The ROC curve yielded an AUC (Area Under Curve) of 0.524, indicating a low level of relationship between these two outcomes. The cutoff value was determined to be 3.02, with a sensitivity of 61.5% and specificity of 52.2% at this value (Table 8, Fig. 12).

**Table 8:** Relation between neck lymph node SUV-Max value and lymph node capsule invasion

	Cut-off	Sensitivity (%)	Specifity (%)	AUC
SUV-Max	3.02	61.5	52.2	0.524

## Conclusion

In some studies in the literature, a statistically significant positive relationship has been observed between SUV-Max levels and perineural invasion parameters. In a study conducted by Dingo and colleagues on patients diagnosed with pancreatic ductal adenocarcinoma, a significant statistical difference was observed in Gallium 68 PET-CT scans between patients with and without perineural invasion [8]. Another study by Dercle et al. found a significant relationship between the condition of perineural invasion and SUV-Max values in patients diagnosed with head and neck carcinoma [9]. Our study also observed a statistically significant relationship between these two parameters. A cutoff value was researched between these two groups as well, and the cutoff was determined to be 13.28. There is no cutoff value obtained for perineural invasion in the studies in the literature.

In a study conducted by Satoshi and colleagues on 356 patients diagnosed with breast cancer, a significant relationship was found between the condition of lymphovascular invasion and SUV-Max levels [10]. Similarly, in another study by Noda and colleagues involving 84 patients with lung adenocarcinoma, a significant relationship between these two parameters was observed, and the cutoff value was determined to be 2.32 [11]. In our study as well, a positive and statistically significant relationship was observed between these two parameters, and the cutoff value was established at 13.28. There is no study in the literature that compares lymphovascular invasion with SUV-Max values in head and neck squamous cell carcinoma (HNSCC).

The relationship between the primary tumor SUV-Max value and neck metastatic lymph node involvement has also been assessed separately. Various studies in the literature, especially aimed at detecting occult lymph node metastasis, are available. A study by Cho and colleagues found a statistically significant relationship between neck metastasis and the primary tumor, even suggesting that the primary tumor SUV-Max value is more effective than the neck lymph node SUV-Max value in terms of neck metastasis [12]. In a study conducted in our country by Gür and colleagues on 36 patients diagnosed with laryngeal SCC, a significant relationship was detected between the primary tumor SUV-Max value and neck metastatic lymph nodes [13]. In another study by Yıldırım and colleagues involving 143 patients with non-small cell lung cancer, no significant relationship was found between lymph node metastasis and primary tumor SUV-Max value [14]. In a study conducted by Yalçınkaya and colleagues on patients diagnosed with lung carcinoma, no positive relationship was observed between the primary tumor SUV-Max value and lymph node metastasis [15]. As for our study, similar to most studies in the literature, a significant relationship was observed between lymph node metastasis and the primary tumor SUV-Max value, with the most clinically effective cutoff value determined to be 14.71.

There are many studies in the literature on the presence of metastatic lymph nodes and lymph node SUV-Max values. Looking at the leading studies, Piotrowicz and colleagues found a very strong relationship between neck lymph node SUV-Max value and the presence of neck metastasis in an evaluation of 216 patients diagnosed with head and neck carcinoma [16]. In another study conducted by Payabvash on 39 patients, a significant difference was observed between benign and malignant lymph nodes in terms of SUV-Max uptake. Demirel and colleagues tested the relationship between the lymph node SUV-Max value cutoff and distant metastasis in 57 patients diagnosed with breast cancer, with the cutoff value determined to be 7.8 [17]. In an extensive study that included 100 lymph nodes in patients with non-small cell lung carcinoma, Köksal and colleagues observed a significant difference between the SUV-Max values of metastatic and non-metastatic lymph nodes. Additionally, on average, patients with squamous cell lung carcinoma had significantly higher metastatic lymph node SUV-Max values than those with adenocarcinoma. The cutoff value for metastatic lymph node SUV-Max was determined to be 5 in all patients diagnosed with non-small cell lung carcinoma [18].

## Results

Our clinical study is the first to evaluate multiple pathological parameters together across multiple locations. Due to the lack of another study that evaluates head and neck cancer pathology results and PET-CT findings on such a comprehensive scale, this work holds significant academic importance. Larger studies conducted on more homogeneous patient groups may yield results with higher sensitivity and specificity, closer to the gold standard, and could provide groundbreaking insights into the management of head and neck cancers, which have various approaches. In particular, there are differing views on prophylactic neck dissection; the SUV-Max value obtained from PET-CT could assist the surgeon in deciding whether to proceed with neck dissection. This would help avoid unnecessary surgeries that carry risks for the patient or the need for a second surgical procedure. The presence of perineural invasion, lymphovascular invasion, multiple lymph node metastases, and lymph node capsular invasion are all indications for postoperative radiotherapy, and informing the patient about these factors is critical in the planning of a multidisciplinary treatment approach. Finally, lymph node capsular invasion and extracapsular spread are among the most important parameters in determining the extent of neck dissection (selective, modified radical, or radical neck dissection). Additionally, these factors are the primary reasons for complications during neck dissection surgery and for postoperative recurrences in the neck. A new and more precise cut-off value for capsular invasion would aid the clinician in deciding on neoadjuvant radiotherapy before surgery.

### The risk of bias assessment

The risk of bias assessment for this retrospective cohort study involved a systematic evaluation of potential sources of bias throughout the research process. This evaluation included consideration of selection bias, information bias, and measurement bias. For selection bias, the study ensured that the inclusion and exclusion criteria were strictly followed and documented. Information bias was mitigated by utilizing standardized and validated data collection methods from the hospital’s automation system. Measurement bias was addressed through the application of consistent PET-CT protocols and the de-identification and objective analysis of data in SPSS. Additionally, confounding variables were controlled by statistical adjustments. The risk of bias was further minimized by the study's retrospective design, which reduces the potential for recall bias and interviewer bias often encountered in prospective studies. The study's conclusions were drawn based on the comprehensive statistical analysis, taking into account the potential impact of identified biases.

## Data Availability

The data supporting the findings of this study are available upon request from authorized hospital personnel, as they are stored in the hospital's internal automation system and are not publicly accessible.

## References

[1] Inoue T, Yutani K, Taguchi T, Tamaki Y, Shiba E, Noguchi S (2004) Preoperative evaluation of prognosis in breast cancer patients by [18F]2-Deoxy-2-fluoro-d-glucose-positron emission tomography. J Cancer Res Clin Oncol 130(5):273–278. 10.1007/s00432-003-0536-5 PMC12161836 14986112

[2] Cho JK, Hyun SH, Choi N, Kim MJ, Padera TP, Choi JY et al (2015) Significance of lymph node metastasis in cancer dissemination of head and neck cancer. Transl Oncol 8(2):119–125 10.1016/j.tranon.2015.03.001 PMC4415144 25926078

[3] Demirel BB, Tosun HE, Uçmak G (2022) The relation between tumor and axillary lymph node SUV values with the presence of distant metastases in staging F18 FDG PET/CT in breast cancer. Acta Oncologica Turcica 55(3):253–260

[4] Bos R, Van der Hoeven JJM, Van der Wall E, Van der Groep P, Van Diest PJ, Comans EFI et al (2002) Biologic correlates of 18fluorodeoxyglucose uptake in human breast cancer measured by positron emission tomography. J Clin Oncol 20(2):379–387 10.1200/JCO.2002.20.2.379 11786564

[5] Dercle L, Hartl D, Rozenblum-Beddok L, Mokrane FZ, Seban RD, Yeh R et al (2018) Diagnostic and prognostic value of 18F-FDG PET, CT, and MRI in perineural spread of head and neck malignancies. Eur Radiol 28(4):1761–1770. 10.1007/s00330-017-5063-x 29086023

[6] Gatta G, Botta L, Sánchez MJ, Anderson LA, Pierannunzio D, Licitra L et al (2015) Prognoses and improvement for head and neck cancers diagnosed in Europe in early 2000s: the EUROCARE-5 population-based study. Eur J Cancer 51(15):2130–2143 10.1016/j.ejca.2015.07.043 26421817

[7] Yıldırım F, Yurdakul A (2018) The relationship between primary tumor metabolic activity and lymph node and distant organ metastasis in non-small cell lung cancer. Article Gazi Med J. 10.12996/gmj.2018.48

[8] Gambhir SS (2002) Molecular imaging of cancer with positron emission tomography. Nat Rev Cancer 2(9):683–93. Available from: https://www.nature.com/articles/nrc882 10.1038/nrc882 12209157

[9] Payabvash S, Meric K, Cayci Z (2016) Differentiation of benign from malignant cervical lymph nodes in patients with head and neck cancer using PET/CT imaging. Clin Imaging 40(1):101–105 10.1016/j.clinimag.2015.09.001 26454617

[10] Piotrowicz O, Jia H, Blazak J (2022) F18-FDG PET/CT accuracy in nodal staging of head and neck squamous cell carcinoma and correlation of SUVmax to the likelihood of a confirmed nodal metastasis. J Med Imaging Radiat Sci. 53(4):599–604. Available from: https://pubmed.ncbi.nlm.nih.gov/36272860/10.1016/j.jmir.2022.09.016 36272860

[11] Gür H, İsmi O, Koç ZP, Koray Bal K, Vayisoğlu Y, Görür K et al (2025) İleri Evre Larinks Kanserlerinde Pozitron Emisyon Tomografi Bulgularının Ameliyat Materyallerindeki Histopatolojik Bulgularla İlişkisi The Relation Between Positron Emission Tomography Findings and Histopathological Findings of Surgical Samples in Advanced Stage Laryngeal Cancers-NC-ND license

[12] McDermott JD, Bowles DW (2019) Epidemiology of head and neck squamous cell carcinomas: impact on staging and prevention strategies. Curr Treat Options Oncol 20(5):1–13. 10.1007/s11864-019-0650-5 31011837

[13] Ding J, Qiu J, Hao Z, Huang H, Liu Q, Liu W et al (2023) Prognostic value of preoperative [68 Ga]Ga-FAPI-04 PET/CT in patients with resectable pancreatic ductal adenocarcinoma in correlation with immunohistological characteristics. Eur J Nucl Med Mol Imaging 50(6):1780–1791. 10.1007/s00259-022-06100-4 36695823

[14] Koksal D, Demirag F, Bayiz H, Ozmen O, Tatci E, Berktas B et al (2013) The correlation of SUVmax with pathological characteristics of primary tumor and the value of Tumor/Lymph node SUVmax ratio for predicting metastasis to lymph nodes in resected NSCLC patients. J Cardiothorac Surg 8(1):1–8. 10.1186/1749-8090-8-63 PMC3622559 23557204

[15] Shiono S, Abiko M, Sato T (2011) Positron emission tomography/computed tomography and lymphovascular invasion predict recurrence in stage I lung cancers. J Thorac Oncol 6(1):43–47 10.1097/JTO.0b013e3181f9abca 21079522

[16] İzmir Göğüs Hastanesi Dergisi (2023) Submission » KÜÇÜK HÜCRELİ DIŞI AKCİĞER KANSERİ OLGULARINDA PET/BT’DEKİ PRİMER TÜMÖR SUVMAX DEĞERİNİN PROGNOSTİK DEĞERİ VE UZAK ORGAN, LENF NODU METASTAZI İLE İLİŞKİSİ. Available from: https://dergipark.org.tr/en/pub/ighd/issue/43144/523160

[17] Tshering Vogel DW, Thoeny HC (2016) Cross-sectional imaging in cancers of the head and neck: How we review and report. Cancer Imaging 16(1):1–15. 10.1186/s40644-016-0075-3 PMC4971750 27487932

[18] Noda Y, Goshima S, Kanematsu M, Watanabe H, Kawada H, Kawai N et al (2016) F-18 FDG uptake on positron emission tomography as a predictor for lymphovascular invasion in patients with lung adenocarcinoma. Ann Nucl Med 30(1):11–17. 10.1007/s12149-015-1023-1 26337532

